# Shenqi Granules Enhance Recovery from Myocardial Ischemia–Reperfusion Injury by Downregulating MMP9 and ADH1C

**DOI:** 10.3390/ph19030475

**Published:** 2026-03-13

**Authors:** Hai-Xin Liu, Xin-Lei Shi, Shu-Yuan Zhou, Yu-Chang Li, Dong-Yan Lin, Pei-You Tan, Zi-Ce Zhou, Ying-Wei Li, Hui-Fang Li, Shi-Yuan Wen

**Affiliations:** 1 College of Traditional Chinese Medicine and Food Engineering, Shanxi University of Chinese Medicine, Jinzhong 030603, China; l-haixin@hotmail.com (H.-X.L.); 18903443858@163.com (X.-L.S.); joooseo_yeon@163.com (S.-Y.Z.); m17636332564@163.com (Y.-C.L.); 15534401488@163.com (D.-Y.L.); tpy917522@163.com (P.-Y.T.); 18369012877@163.com (Z.-C.Z.); y13393545682@outlook.com (Y.-W.L.); 2 College of Basic Medical Sciences, Shanxi Medical University, Jinzhong 030606, China

**Keywords:** myocardial ischemia–reperfusion injury, Shenqi granules, MMP9, ADH1C, immunity

## Abstract

**Background:** Shenqi granule (SQG) was used clinically to strengthen the spleen and boost energy, alleviating physical weakness and limb fatigue caused by energy deficiency. However, the specific effects and potential molecular mechanisms of SQG in myocardial infarction (MI) treatment remain to be clarified. **Methods:** This study thoroughly evaluates SQG’s role in improving MIRI in rats using a biological approach. Network pharmacology, weighted gene co-expression network analysis (WGCNA), receiver operating characteristic (ROC), and immune landscape analysis were used to analyze components and key molecular targets. The therapeutic targets of SQG were then validated through molecular docking, molecular dynamics simulation, and experiments. **Results:** SQG reduced myocardial infarct size and improved myocardial function in rats. Network pharmacology analysis found that six bioactive compounds in SQG could target four proteins. Using WGCNA and ROC, two key targets of SQG were identified, MMP9 and ADH1C. Importantly, integrating PPI network prediction, molecular docking, and expression correlation analyses, MMP9 and ADH1C demonstrate strong physical binding potential and expression association, suggesting their possible involvement in MIRI-related pathways through the immune microenvironment. Molecular experiments and other methods confirmed that the five active ingredients in SQG (luteolin, quercetin, hederagenin, 7-O-methylisomucronulatol, and stigmasterol) can exert cardioprotective effects by stably binding to MMP9/ADH1C. **Conclusions:** SQG reduces myocardial infarct volume and enhances myocardial function in MIRI rats, likely via inhibiting MMP9 and ADH1C expression. This suggests SQG’s potential as a therapeutic agent for MI, with findings offering strong scientific support for SQG’s use in cardiovascular disease research.

## 1. Introduction

Myocardial infarction (MI) caused by coronary artery thrombotic occlusion is a leading global cause of morbidity and mortality [1]. While percutaneous coronary intervention (PCI) is the optimal and most effective treatment for restoring coronary artery blood flow swiftly, it can paradoxically lead to additional irreversible myocardial injury and cell death, termed myocardial ischemia/reperfusion injury (MIRI) [2]. Despite advances in care, there are no established clinical therapies to effectively mitigate MIRI. Consequently, even with timely and complete revascularization, the 1-year mortality post-myocardial infarction remains at 7%, soaring to 22% for patients who develop heart failure [3]. Thus, MIRI is still a key target for the treatment of MI, and it is necessary to further explore the effective therapeutic drugs and potential targets of MIRI.

The pathophysiological processes that cause MIRI are not fully understood. Thrombosis and inflammation are critical in the pathophysiology of MIRI [4]. Evidence, including our own, increasingly indicates that immune responses play a central role in MIRI, characterized by the recruitment and activation of immune cells linked to both the innate and adaptive immune systems [5]. Immune cells such as neutrophils, monocytes, and dendritic cells are rapidly recruited to the infarcted area. They initiate inflammatory responses and clear necrotic tissue [6]. Matrix metalloproteinase-9 (MMP-9) is a key factor during MIRI [7]. It drives inflammation by degrading the extracellular matrix and activating pro-inflammatory factors like IL-1β, IL-8, and CXCL6, creating a positive feedback loop that sustains inflammation [8]. Also, MMP-9 promotes the aggregation and chemotaxis of white blood cells, prolonging the inflammatory phase [9]. Recently, the genetic polymorphism of alcohol dehydrogenase type 1C (ADH1C) has been associated with a lower risk of MI [10], but the role and mechanisms of ADH1C in MIRI require further research.

In recent years, traditional Chinese medicine (TCM) has proven to be a valuable resource for developing new MI drugs [11]. In China, TCM has long been used to treat MI, characterized by its multi-component, multi-target and multi-pathway approach [12]. Network pharmacology and experimental validation demonstrate that Buyang Huanwu Decoction protects against MIRI through 95 active components targeting 75 disease-related genes, with key hubs including IL-6, AKT1, TNF, ICAM1, VCAM1, MMP9, and IL-10 enriched in TNF signaling, apoptosis, and p53 pathways [13]. In rat MIRI models, the formula reduces infarct size by suppressing inflammatory adhesion cascades and MMP9-mediated matrix degradation while activating AKT1-mediated survival signaling [13]. Similarly, Yixinshu (derived from Shengmai San) attenuates infarction, oxidative/nitrative stress, and mitochondrial apoptosis while upregulating LXRα, suggesting that TCM formulas mitigate reperfusion injury through coordinated modulation of lipid metabolism and nuclear receptor pathways [14].

The “Shenqi” prescription, derived from Shicheng Gu’s “Yangyi Daquan” during the Qing Dynasty, consists of two main herbs, *Astragalus membranaceus* Fisch. ex Bunge (Huangqi) and *Codonopsis pilosula* Nannf. (Dangshen), and these plant names have been checked with World Flora Online. Today, it has been refined into Shenqi granules (SQGs), used clinically to strengthen the spleen and boost energy, alleviating physical weakness and limb fatigue caused by energy deficiency. However, the specific effects and potential molecular mechanisms of SQG in MI treatment remain to be clarified. This study seeks to investigate the therapeutic effects and mechanisms of SQG on ischemia–reperfusion (I/R) injury utilizing a MIRI rat model. The findings will provide robust scientific evidence supporting the application of SQG in cardiovascular disease research.

## 2. Results

### 2.1. SQG Alleviates MIRI Infarct Volume in Rats

**Figure 1:**
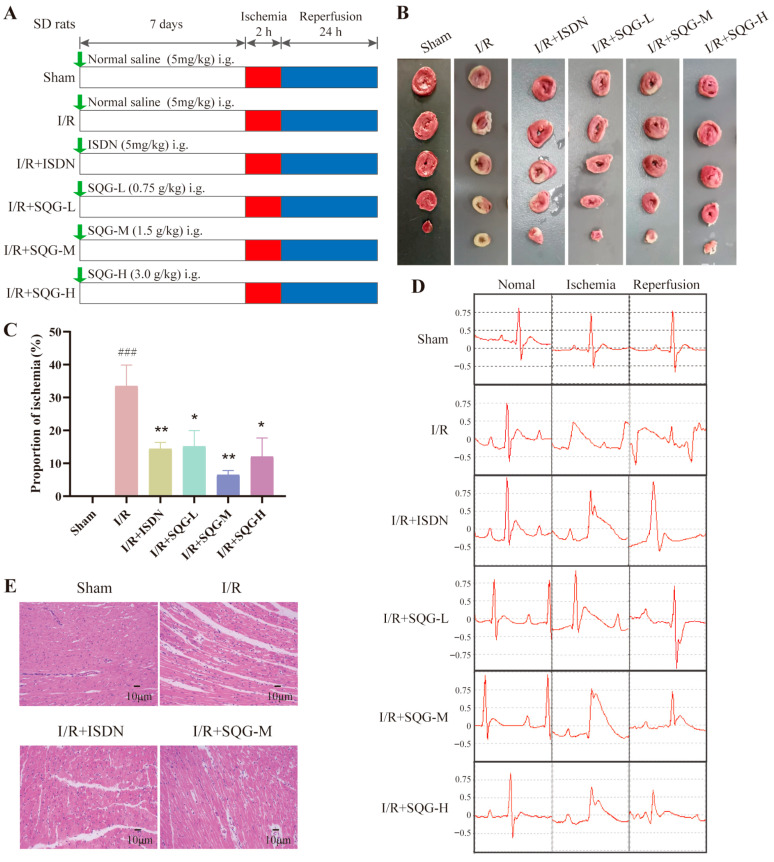
**SQG alleviates myocardial infarct volume in MIRI rats.** (**A**) Timeline for constructing the animal model and treatment. (**B**) Myocardial infarct volume across groups. (**C**) Statistical analysis of infarct volume across groups. (**D**) ECG analysis of MIRI rats. (**E**) HE staining of myocardial tissue. *n* = 10 rats per group. Data are presented as mean ± SEM. ### *p* < 0.001, compared with the Sham group. * *p* < 0.05, ** *p* < 0.01, compared with the I/R model group.

To assess SQG’s potential impact on MIRI, we induced MIRI in 8-week-old male SD rats. Figure 1A outlines the timeline for animal model construction and drug treatment. TTC staining quantified myocardial infarction severity, with Figure 1B showing infarct size differences among groups. Compared to the I/R model, positive drug ISDN and all SQG doses significantly reduced infarct size (*p* < 0.05, Figure 1C), with the medium SQG dose being most effective. Subsequent experiments thus used this dose. ECG results indicated that SQG improved cardiac function (Figure 1D). HE staining of myocardial tissue revealed disordered myofibers and large transparent areas due to edema in the I/R model group, compared to the Sham group. The medium-dose SQG group showed significant improvements in myofiber disorder and edema (Figure 1E). Overall, SQG demonstrated beneficial effects on MI.

### 2.2. Potential Ingredients and Targets Were Screened for SQG Treatment of MIRI

**Figure 2:**
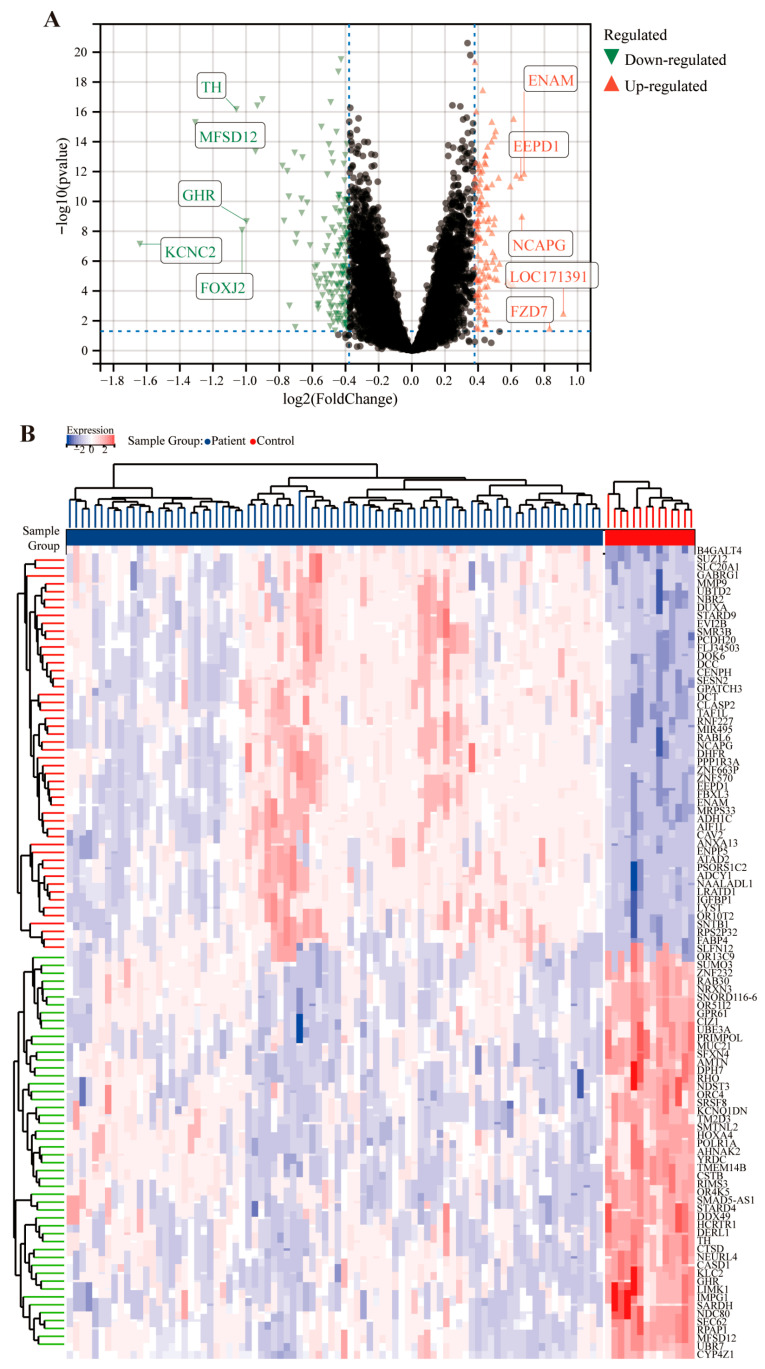
**MI targets were analyzed.** (**A**) The volcano plot shows significant DEGs of MI, with the x-axis representing log_2_(FoldChange) and the y-axis representing −log_10_(*p* value). Green inverted triangles denote significantly down-regulated genes, red triangles denote significantly up-regulated genes, and black dots represent genes with no significant differential expression. The vertical blue dashed lines indicate the fold-change threshold of |log_2_(FoldChange)| = 0.4, and the horizontal blue dashed line indicates the significance threshold of −log_10_(*p* value) = 1.3 (corresponding to *p* = 0.05). (**B**) The heatmap of DEGs associated with MI.

The GEO database was used to analyze potential targets of SQG in improving MIRI. In the MI dataset GSE62646, samples were divided into control and patient groups. DEGs were identified between the two groups using a threshold of fold change 1.3 and adjusted *p*-value < 0.05, yielding 275 DEGs (114 upregulated and 161 downregulated) (Figure 2A). A heatmap displayed their expression levels across samples (Figure 2B).

**Figure 3:**
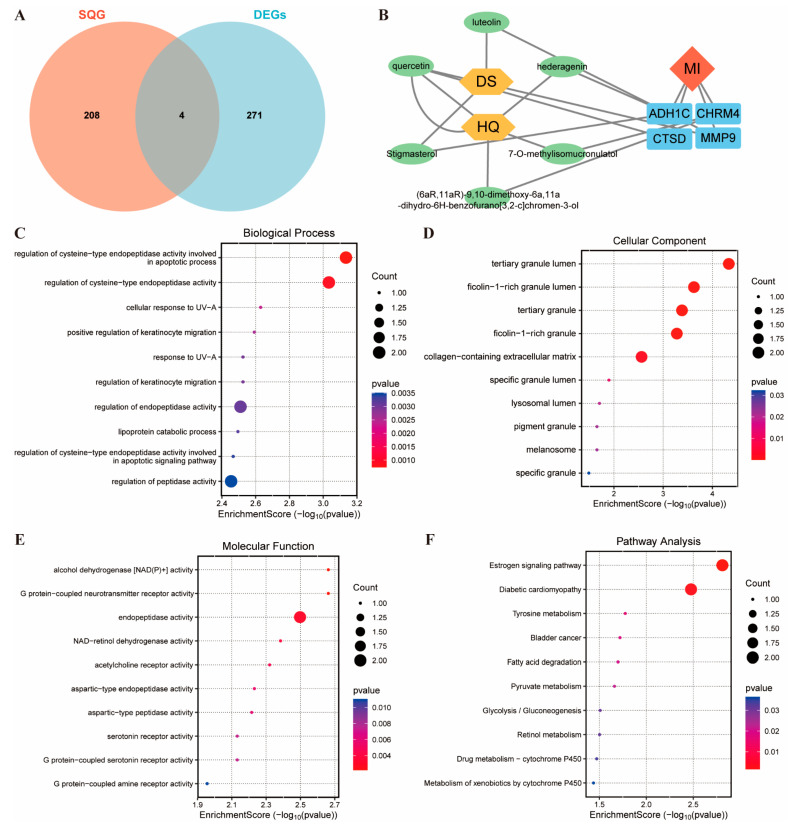
**Component–disease interaction maps were constructed using network pharmacology methods.** (**A**) Venn diagram for the intersectional genes between active ingredient targets and DEGs of MI. (**B**) Cytoscape (Version 3.10.1) network map between active ingredients and DEGs. (**C**) Biological process based on GO analysis. (**D**) Cellular component based on GO analysis. (**E**) Molecular function based on GO analysis. (**F**) KEGG pathway analysis.

Chemical components of Astragalus membranaceus and Codonopsis pilosula were screened based on OB ≥ 30% and DL ≥ 0.18, obtaining 43 bioactive compounds (Table S1 in the Supplementary Materials). Extracting the targets of these compounds gave 212 genes. Venn analysis found an overlap of 4 genes (ADH1C, CHRM4, MMP9, CTSD) between the compound targets and disease-related DEGs (Figure 3A). A Sankey diagram linked traditional Chinese medicine, bioactive compounds and these 4 genes, suggesting that 6 SQG components (luteolin, quercetin, hederagenin, 7-O-methylisomucronulatol, stigmasterol, (6aR,11aR)-9,10-dimethoxy-6a,11a-dihydro-6H-benzofurano[3,2-c]chromen-3-ol) may improve MI by acting on these genes (Figure 3B).

GO enrichment analysis of these 4 genes showed that in the biological process (BP) category, the main terms were regulation of cysteine-type endopeptidase activity (related to apoptosis and signaling), regulation of endopeptidase activity, and regulation of peptidase activity (Figure 3C). For the cellular component (CC), the main terms were collagen-containing extracellular matrix, two granule lumens (tertiary granule lumen and ficolin-1-rich granule lumen), and two lumens (tertiary granule and ficolin-1-rich granule, Figure 3D). For molecular function (MF), the main terms were endopeptidase activity (Figure 3E). KEGG pathway analysis highlighted the estrogen signaling pathway and diabetic cardiomyopathy (Figure 3F).

### 2.3. The Core Targets Related to MIRI Were Identified by WGCNA

**Figure 4:**
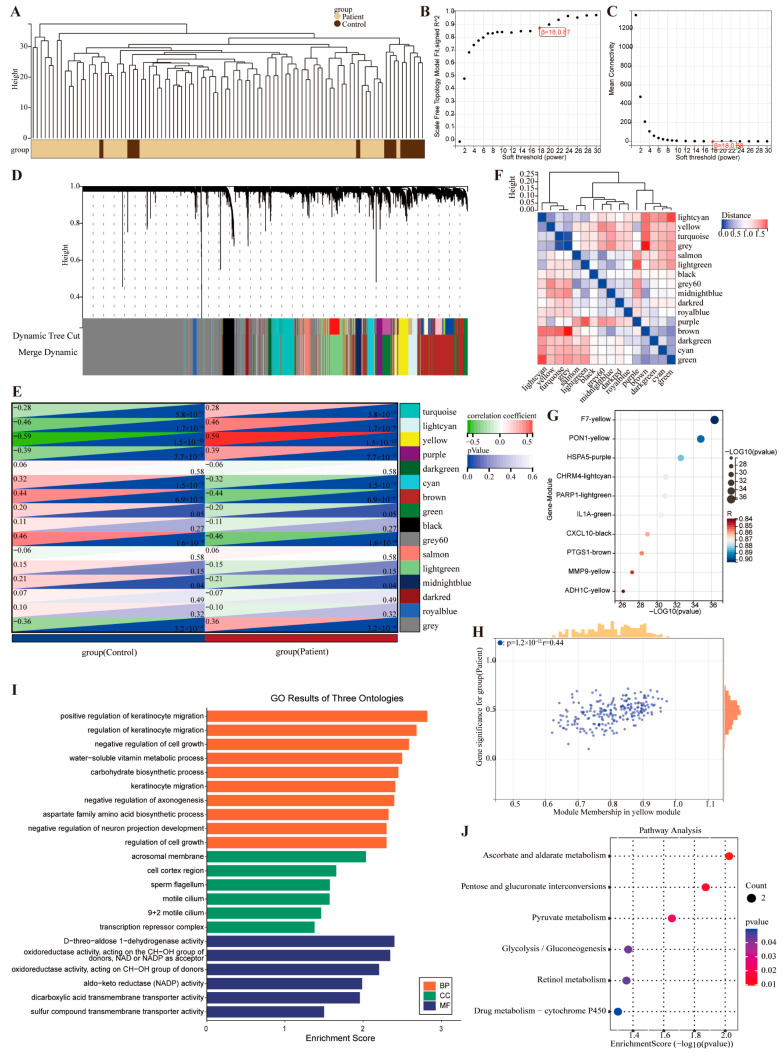
**WGCNA identified MI-related co-expressed gene modules.** (**A**) Sample clustering analysis based on the MI-related GSE62646 dataset. (**B**) Analysis of the scale-free fitting index for various soft-thresholding powers β. (**C**) Analysis of the average connectivity for various soft-thresholding powers β. (**D**) Hierarchical clustering dendrogram of co-expression genes, where each leaf on the dendrogram corresponds to a gene module and each color represents a gene module. (**E**) Heatmap of the Pearson correlation coefficients between the modules and the MI phenotype, with each cell containing the corresponding correlation coefficient and *p*-value. (**F**) Modules and inter-module correlation. (**G**) Bubble plot of the top 10 DEGs. (**H**) Scatter plot of gene significance versus module membership (MM) for the genes in the yellow module. (**I**) GO enrichment analysis of the yellow module genes. (**J**) KEGG pathway analysis of the yellow module genes.

WGCNA was employed to identify the core targets of MIRI. Sample-level clustering was performed using the “WGCNA” package in R (Figure 4A). Topological calculations determined a soft threshold β = 18 as optimal for constructing a scale-free network (Figure 4B,C). We have extracted the scale-free goodness-of-fit R^2^ value of 0.87 at β = 18 from the original WGCNA output, which satisfies the established criterion for scale-free network topology (R^2^ > 0.8), demonstrating that the co-expression network constructed in this study exhibits robust scale-free characteristics. This threshold transformed the correlation matrix into an adjacency matrix and then into a TOM. Hierarchical clustering with average linkage grouped relevant modules, each containing at least 30 genes (Figure 4D). During module division, the sensitivity parameter was set to 3 to optimize clustering. The dissimilarity of module eigengenes was calculated. By setting the module dendrogram cut line and merging modules with a distance < 0.25, 16 co-expression modules were obtained (Figure 4E). The grey module comprised genes unassigned to any specific module (Figure 4E). The correlation between and within modules is shown in Figure 4F. For the 15 modules, the bubble chart displays clinically significant modules and highly connected genes (Figure 4G). Using |MM| > 0.8 as the threshold, the yellow module showed the strongest correlation with MIRI (r = 0.59, *p* = 1.5 × 10^−10^) and was identified as the core module. Within the yellow module, 117 high-connectivity hub genes were identified as key targets for subsequent analysis. The correlation between gene significance (GS) and MM values of genes in the yellow module validated the hub gene screening (Figure 4H).

GO and KEGG enrichment analyses were then performed on the yellow module genes. In GO analysis, BP terms mainly involved positive regulation of keratinocyte migration, CC terms were enriched in the acrosomal membrane, and MF terms primarily involved D-threo-aldose 1-dehydrogenase activity (Figure 4I). KEGG analysis highlighted Ascorbate and aldarate metabolism, Pentose and glucuronate interconversions, and Pyruvate metabolism (Figure 4J), suggesting the targets might be the regulation of endogenous substance metabolism.

### 2.4. MMP9 and ADH1C Play Important Roles in the Alleviation of MIRI by SQG

**Figure 5:**
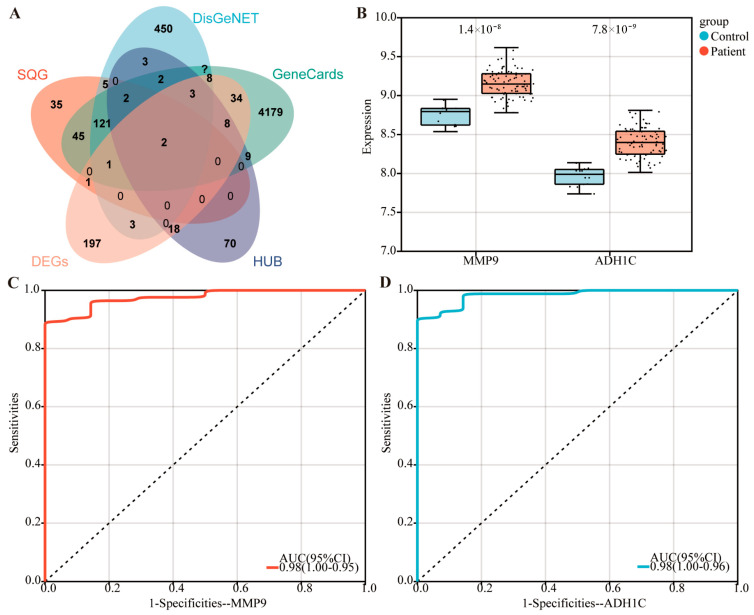
**SQG targets were screened for the treatment of MIRI.** (**A**) Venn diagram for the intersectional genes from the hub gene of yellow module, SQG targets, GeneCards, DisGeNET, and DEGs. (**B**) The expression analysis of MMP9 and ADH1C in GSE62646 dataset. (**C**,**D**) The ROC analysis of MMP9 (**C**) and ADH1C (**D**).

We delved deeper into the key targets of SQG in alleviating MIRI. Using “myocardial infarction” as the keyword, we searched GeneCards and DisGeNET for disease-related targets. By intersecting targets from SQG, DEGs, hub, GeneCards, and DisGeNET, two critical disease-associated genes were pinpointed, MMP9 and ADH1C (Figure 5A). CHRM4 and CTSD genes were analyzed through WGCNA and were not incorporated into the core module (yellow module), which is highly associated with MIRI, so these genes are not hub genes. In the GSE62646 dataset, both genes showed significant upregulation in the disease group versus the normal group (Figure 5B). ROC analysis revealed an AUC of 0.98 for both genes (Figure 5C,D), highlighting their crucial role in SQG’s mitigation of MIRI. Thus, MMP9 and ADH1C are likely key targets for SQG in alleviating MIRI.

### 2.5. MMP9 and ADH1C Are Crucial in Immune Infiltration During MIRI

**Figure 6:**
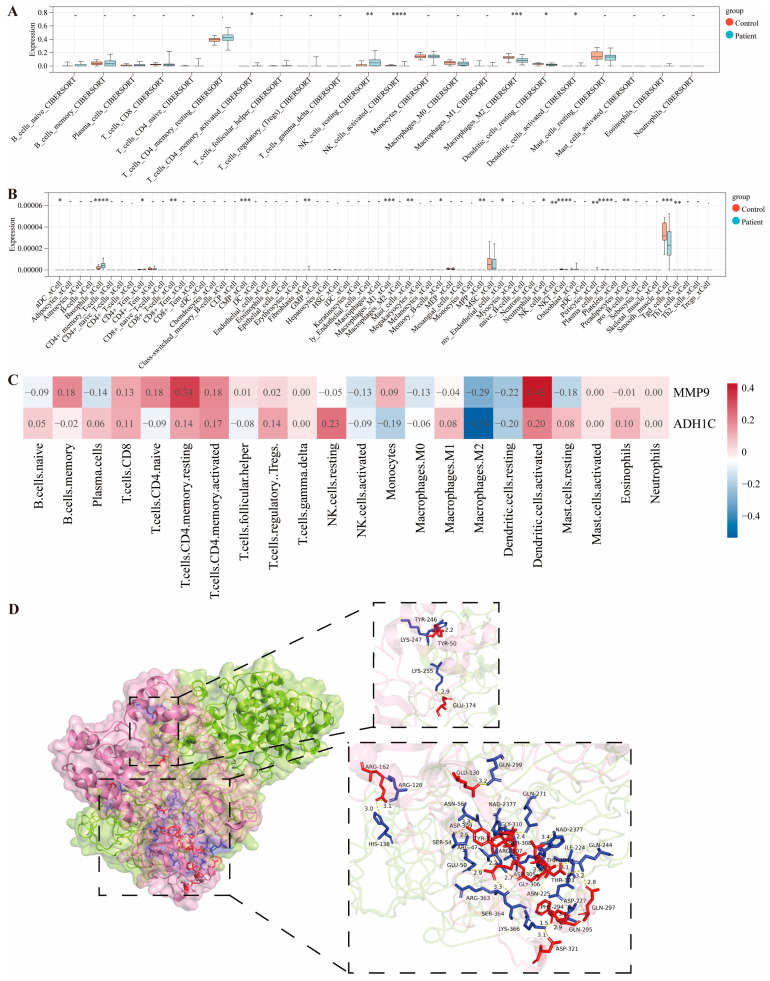
**MMP9 and ADH1C play important roles in immune infiltration of MIRI.** (**A**,**B**) The differences in immune cell infiltration between patients and controls were analyzed using CIBERSORT (**A**) and Xcell (**B**) algorithms. (**C**) Correlation analysis between immune cells and MMP9 or ADH1C. (**D**) Predictive analysis of MMP9 protein and ADH1C protein interaction. * *p* < 0.05, ** *p* < 0.01, *** *p* < 0.001, **** *p* < 0.0001, compared with the control group.

To analyze immune cell infiltration in MI patients versus normal controls, both CIBERSORT and Xcell algorithms were employed, with rank-sum tests identifying significantly different cell subsets between the groups. CIBERSORT analyzed 22 immune cell subsets and found 6 with significant differences (*p* < 0.05, Figure 6A). Activated CD4+ memory T cells were enriched in patients (*p* = 0.03), indicating possible activation of specific T-cell-mediated immune responses, worsening local inflammation. Resting NK cells were elevated (*p* = 3.1 × 10^−3^) while activated NK cells were reduced (*p* = 4.0 × 10^−5^) in patients, suggesting NK cell functional imbalance, which may affect immunosurveillance and cytotoxic clearance in ischaemic myocardium. M2 macrophages were decreased (*p* = 2.6 × 10^−4^) in patients, implying insufficient reparative macrophages and potential repair delays. Both resting and activated dendritic cells showed significant differences (*p* = 0.03 and *p* = 0.04, respectively), indicating altered antigen-presenting cell activation, contributing to ischaemia-induced immune activation.

Xcell analyzed immune and stromal cell infiltration and detected nine significantly different cell subsets (*p* < 0.05, Figure 6B). Basophils were increased (*p* = 4.0 × 10^−4^) in patients, possibly enhancing vascular permeability and inflammation. CD8+ T cells were enriched (*p* = 0.03), indicating higher cytotoxic T-cell infiltration, which may contribute to myocardial apoptosis or damage. Myeloid progenitor cells were accumulated (*p* = 0.02), while M1 macrophages showed a decreasing trend (*p* = 9.6 × 10^−3^), reflecting myeloid cell differentiation and inflammatory imbalance. Fibroblasts were elevated (*p* = 0.03), suggesting ischaemia-induced fibrotic microenvironment activation, whereas smooth muscle cell infiltration was reduced (*p* = 8.4 × 10^−3^), possibly related to vascular remodeling. Plasma cells and NKT cells also differed significantly (*p* = 5.6 × 10^−4^ and *p* = 8.1 × 10^−4^, respectively), indicating dysregulated humoral and innate immunity in MI pathogenesis.

To explore the relationship between the core genes MMP9 and ADH1C and immune cell infiltration, correlation analyses were performed. The heatmap revealed significant heterogeneity in correlations between these genes and immune cell infiltration, with modular distributions of positive (red) and negative (blue) correlations (Figure 6C). MMP9 showed the strongest positive correlation with activated dendritic cells, while ADH1C showed the strongest negative correlation with M2 macrophages. Additionally, interaction prediction analysis of MMP9 and ADH1C showed a docking score of −235.79 and a confidence score of 0.8476, indicating strong protein–protein interactions with multiple hydrogen bonds (Figure 6D). Thus, MMP9 and ADH1C may regulate the MIRI immune microenvironment.

### 2.6. SQG Improves MIRI by Reducing the Expression of MMP9 and ADH1C

**Figure 7:**
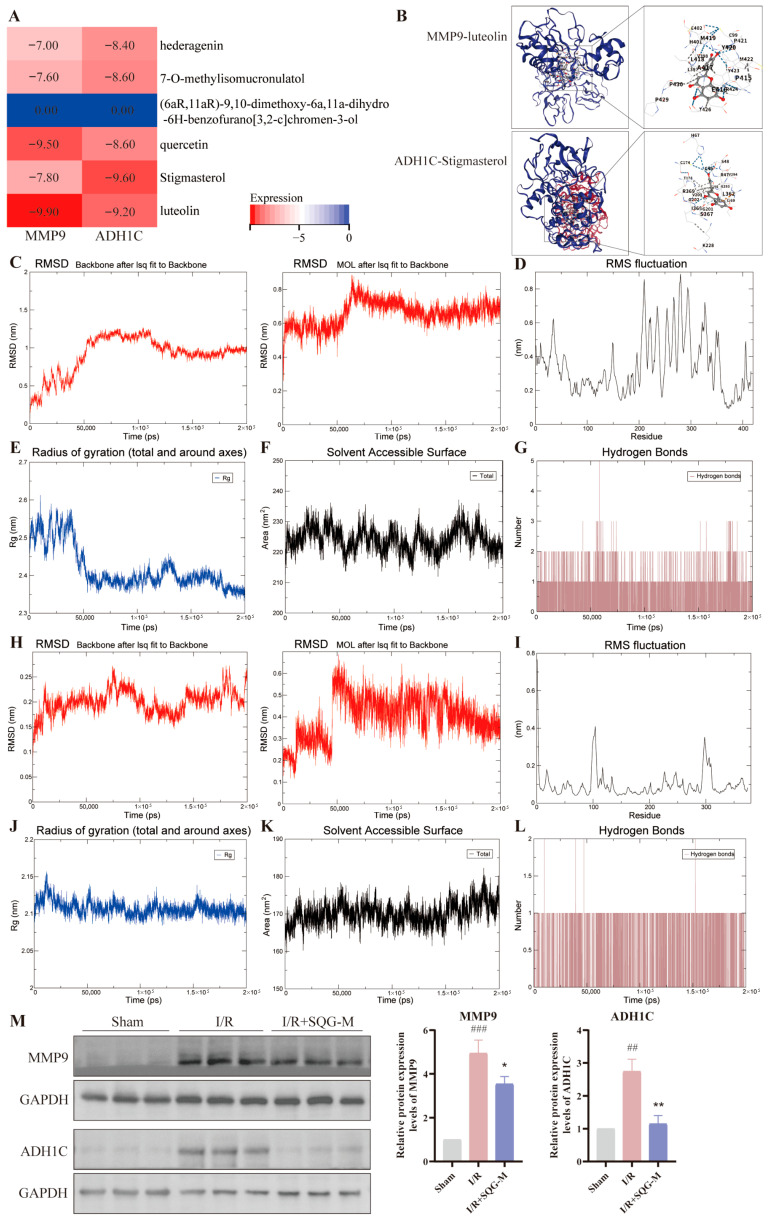
**MMP9 and ADH1C are the key targets of SQG improving MIRI.** (**A**) Heatmap of binding energies between six SQG components and MMP9/ADH1C. (**B**) Molecular docking: MMP9-luteolin and ADH1C-stigmasterol. (**C**–**G**) Molecular dynamics of the MMP9-luteolin complex: system RMSD (**C**), RMSF (**D**), Rg (**E**), SASA (**F**), and hydrogen bonds (**G**). (**H**–**L**) Molecular dynamics of the ADH1C-stigmasterol complex: system RMSD (**H**), RMSF (**I**), Rg (**J**), SASA (**K**), and hydrogen bonds (**L**). (**M**) Western blot results for MIRI treatment with SQG, *n* = 3 rats per group. Data are presented as mean ± SEM of three independent experiments. ## *p* < 0.01, ### *p* < 0.001, compared with the Sham group. * *p* < 0.05, ** *p* < 0.01, compared with the I/R model group.

The six components in SQG may act on MIRI, and MMP9 and ADH1C are likely key targets for SQG in alleviating MIRI, so we analyzed whether there were any interactions between these six components and these two targets. Blind docking of six components related to MMP9 and ADH1C predictions showed that, except for (6aR,11aR)-9,10-dimethoxy-6a,11a-dihydro-6H-benzofurano[3,2-c]chromen-3-ol, which showed no binding to either MMP9 or ADH1C, the other components (luteolin, quercetin, hederagenin, 7-O-methylisomucronulatol, and stigmasterol) had docking scores of less than −6 (Figure 7A). The lowest docking score was for MMP9 with luteolin, followed by ADH1C with Stigmasterol, and their molecular docking is shown in Figure 7B. Molecular dynamics analyses were then performed on the MMP9-luteolin and ADH1C-stigmasterol complexes.

RMSD analysis indicated stable binding between MMP9 and luteolin (Figure 7C). RMSF results showed high RMSF values for most residues, indicating a flexible protein structure (Figure 7D). The Rg values decreased and stabilized, suggesting a more compact MMP9 structure upon luteolin binding (Figure 7E). The SASA plot showed fluctuations, revealing stable Solvent Accessible Surface Area changes and dynamic stability in the MMP9-luteolin binding (Figure 7F). Stable hydrogen bond numbers indicated strong interactions between MMP9 and luteolin (Figure 7G). Overall, the molecular dynamics simulation showed stable binding between MMP9 and luteolin.

RMSD analysis showed stable binding between ADH1C and stigmasterol (Figure 7H). RMSF results revealed low RMSF values for most residues, indicating a stable protein structure (Figure 7I). The fluctuation of the Rg value is very small, suggesting stable ADH1C and stigmasterol binding (Figure 7J). The SASA plot displayed fluctuations, revealing steady Solvent Accessible Surface Area changes and dynamic stability in the ADH1C-stigmasterol binding (Figure 7K). Stable hydrogen bond numbers indicated strong interactions between ADH1C and Stigmasterol (Figure 7L). The molecular dynamics simulation thus confirmed stable binding between ADH1C and Stigmasterol.

Western blot results showed that MMP9 and ADH1C protein expression were increased after myocardial I/R compared with the Sham group; however, compared with the I/R model group, MMP9 and ADH1C protein expression were significantly decreased in the SQG group (Figure 7M). Thus, SQG improved MIRI by reducing MMP9 and ADH1C expression.

## 3. Discussion

SQG has been shown to reduce myocardial infarct size and enhance myocardial function in MIRI rats. Network pharmacology analysis has identified six bioactive compounds in SQG that are key to improving MIRI. It is likely that SQG achieves these benefits by targeting MMP9 and ADH1C. Five SQG components (luteolin, quercetin, hederagenin, 7-O-methylisomucronulatol, and stigmasterol) can bind to these two targets. Importantly, SQG treatment reduces MMP9 and ADH1C expression in the myocardial tissue of MIRI rats. Thus, SQG promotes recovery from MIRI by downregulating MMP9 and ADH1C.

Astragalus and its components, such as Astragalus polysaccharides, astragalosides and Astragalus flavonoids, have been reported to protect against myocardial injury from I/R [15]. Astragalus can restore stunned myocardial cells, lower heart rate, reduce cardiac enzyme release, protect vascular endothelial cells, limit myocardial ischaemia, and improve left ventricular diastolic and systolic function after I/R. It scavenges oxygen free radicals, inhibits lipid peroxidation, and shields myocardial cells from oxidative stress. Astragalus also helps reduce calcium overload and maintain intracellular calcium homeostasis, preventing calcium-induced myocardial injury. By inhibiting the generation and release of inflammatory factors, it mitigates inflammatory damage to the myocardium [16]. Codonopsis and its components have been less reported in the context of MI. However, it has been found that Codonopsis extracts can enhance cardiac differentiation and improve cardiac function in infarcted rats [17]. Quercetin, a flavonoid found in many fruits and vegetables, has been shown to have protective effects against MI and MIRI through various mechanisms [18]. Importantly, long-term dietary enrichment with 0.2% quercetin alleviated MMP9 expression and dystrophin cardiac pathology in Mdx/Utrn+/−mice [19]. Luteolin alleviates MIRI through the Siti1/NLRP3/NF-κB pathway [20] and reduces MMP9 level to improve cardiac injury [21]. Hederagenin protects against MIRI by restraining ferroptosis [22]. Stigmasterol has been reported to be cardioprotective by attenuating doxorubicin-induced cardiotoxicity [23]. However, another study revealed that stigmasterol accumulation could cause cardiac damage and promote mortality [24]. There have been no studies on the role of 7-O-methylisomucronulatol in cardiovascular diseases.

We attribute the weaker efficacy of SQG-H (3.0 g/kg) versus SQG-M (1.5 g/kg) to the “optimal dose window” inherent to an over-the-counter product. The human labeled dose was established by toxicology panels that excluded supra-therapeutic multiples; in rats, 3.0 g/kg represents twice the clinically translated medium dose and may exceed the optimal window for individual constituents. Specifically, stigmasterol, one of the major phytosterols in SQG, has been reported to evoke mild cardiomyocyte oxidative stress when present in excess, thereby blunting the protective synergy of luteolin and quercetin. This self-limiting effect reinforces the rationale for selecting the medium (clinically equivalent) dose as the most efficient intervention. It should be emphasized that SQG-H still reduced infarct size relative to I/R (*p* < 0.05, Figure 1C) and never became harmful; consequently, the central conclusion that “SQG ameliorates MIRI” remains fully valid.

The immune microenvironment in myocardial ischaemia shows an imbalance between pro-inflammation and repair. Activated T-cell subsets (like CD4+ memory T cells and CD8+ T cells) and dendritic cells may intensify local inflammation and drive myocardial cell apoptosis. Reduced M2 macrophages and suppressed resting NK cells weaken the body’s ability to repair ischaemic tissue and clear out damaged cells. Increased fibroblast infiltration points to active fibrosis, and decreased smooth muscle cells may affect vascular stability, both contributing to ischaemic cardiomyopathy. These findings reveal potential targets for myocardial ischaemia immunotherapy, such as boosting M2 macrophage polarization, enhancing NK cell function, or curbing T-cell activation pathways, offering new ways to improve the ischaemic myocardial microenvironment.

After a heart attack, necrotic myocardial cells release damage-associated molecular patterns, activating the innate immune response [25]. Immune cells like neutrophils, monocytes, and dendritic cells are quickly recruited to the infarct area, triggering inflammation and clearing dead tissue. Then, macrophages switch to an anti-inflammatory type (type 2 macrophage), secreting anti-inflammatory cytokines such as IL-10 and TGF-β, which promote granulation tissue formation and myocardial repair [26]. Also, resident macrophages can limit monocyte recruitment, reducing inflammation and improving heart remodeling [27]. Dendritic cells (DCs) accumulate at the damage site, presenting antigens in mediastinal lymph nodes to activate T cells [28]. Plasmacytoid DCs (pDCs) mainly produce type I interferons to drive inflammation, while conventional DCs (cDCs) help recruit regulatory T cells (Tregs) to aid repair [29,30]. Adaptive immune cells, such as T cells, also play roles post-MI. CD4+ T cells assist in wound healing, whereas CD8+ T cells may cause myocardial cell apoptosis and worsen heart function by releasing substances like granzyme B [31,32,33]. Tregs can suppress inflammation and enhance myocardial repair [34]. NK cells may facilitate myocardial healing by aiding in the formation of new blood vessels, thereby enhancing the blood supply to the heart muscle and playing a crucial role in recovery following a heart attack [35]. In summary, immune cells play diverse roles after a heart attack, contributing to both inflammatory responses, which clear necrotic tissue, and cardiac repair and remodeling through polarization and factor secretion. Exploring these mechanisms can identify effective therapeutic targets and enhance post-MI recovery.

MMP-9 plays a key role in MIRI. In the early stages of MIRI, neutrophils are the primary source of MMP-9 [36]. They begin to infiltrate a few minutes after MI, peak at 1–3 days, and release MMP-9 from gelatinase granules when activated [37,38]. MMP-9 degrades the extracellular matrix (ECM), increases cardiac microvascular permeability, releases ECM-bound chemokines, and exposes ECM sites with chemotactic properties (such as matrix cryptic sites), promoting white blood cell migration to the infarcted area [39]. It also drives monocyte and macrophage accumulation and chemotaxis, prolonging the inflammatory phase and worsening myocardial damage [40]. Macrophages are another rich source of MMP-9. Significant macrophage infiltration is seen 3 days after infarction, peaking at day 5 [37]. Depending on the stimulating signals, macrophages can take on a pro-inflammatory M1 phenotype or an anti-inflammatory M2 phenotype. M1 macrophages dominate on day 1, while M2 macrophages become dominant from day 3. M1 macrophages secrete MMP-9 to degrade the ECM, while M2 macrophages secrete growth factors, regulate fibroblast differentiation, and stimulate ECM production and deposition for scar formation [37,41]. MMP-9, a marker of M1 macrophages, also processes inflammatory molecules like CXCL4, IL-8, CXCL-12, and TGF-β1, promoting M2 polarization and aiding tissue repair and regeneration in the late inflammatory phase. MMP-9 levels rise early after infarction and remain elevated in the first week, in line with white blood cell infiltration [37]. This indicates that white blood cells are the main source of MMP-9 post-infarction. By degrading the ECM and activating pro-inflammatory factors such as IL-1β, IL-8, and CXCL6, MMP-9 forms a positive feedback loop that sustains inflammation [41,42]. Its overactivation can lead to myocardial cell apoptosis and worsening heart function [43]. Thus, regulating MMP-9 activity is a potential therapeutic strategy for MIRI.

Few studies have explored the link between ADH1C and MIRI. ADH1C’s enzyme activity may affect myocardial energy metabolism and oxidative stress, influencing MIRI development. Its genetic polymorphism may alter individual MIRI susceptibility, with certain genotypes possibly tied to lower MI risk, likely due to differing inflammatory and oxidative stress responses [10]. Also, ADH1C might regulate ethanol metabolism, impacting myocardial cell antioxidant capacity and inflammation [43]. However, the exact mechanisms remain unclear. Overall, ADH1C’s role in MIRI warrants further study, and related research might reveal new MIRI treatment targets. Similarly, little is known about ADH1C’s interplay with immunity in MIRI. While no direct link is established, ADH1C may affect ROS levels modulating inflammation [44], implying it may enhance myocardial cell antioxidant capacity, reduce oxidative stress, suppress inflammatory factor release, and protect myocardial cells from damage.

As emphasized by Lee et al. (2025) in their PathNetDRP framework, PPI connections and network co-pathway enrichment can only suggest potential functional associations between genes and cannot be equated with authentic synergistic biological functions [45]. Integrating PPI network prediction, molecular docking, and expression correlation analyses, MMP9 and ADH1C demonstrate strong physical binding potential and expression association, suggesting their possible involvement in MIRI-related pathways through the immune microenvironment. However, direct experimental evidence for their synergistic biological function in the MIRI immune microenvironment is currently lacking. The core pathological process of MIRI has been systematically characterized, wherein damage-associated molecular patterns (DAMPs) activate innate immunity and trigger massive immune cell infiltration, forming a complex immune microenvironment that provides contextual support for the involvement of both genes in immune regulation [46]. MMP9 has been established as an immune–inflammation-related hub node through network meta-analysis, participating in inflammatory amplification and extracellular matrix remodeling and thereby playing a critical role in the immune microenvironment [47]. Furthermore, the coupling mechanism between immune dysregulation and metabolic abnormalities in MIRI has been revealed, offering pathological logic for the potential association between ADH1C (a metabolism-related enzyme) and MMP9 (an immune–inflammatory molecule) [48,49].

Myocardial fibrosis plays an important role in MIRI. The administration protocol in this study involved intragastric dosing beginning 7 days prior to ischemia modeling and continuing for 7 days, with administration maintained for 24 h after ischemia–reperfusion prior to animal euthanasia. This preventive plus acute-phase dosing paradigm aligns with the long-term medication characteristics of coronary heart disease patients in clinical practice, rather than immediate post-IR injury administration. The study focused on myocardial injury repair, inflammatory response modulation, and core target expression changes during the acute phase of MIRI (within 24 h). Myocardial fibrosis, however, represents a long-term pathological consequence of IR injury, typically requiring 1–4 weeks post-surgery for evident collagen deposition and fibrotic remodeling. Within this 24 h short-term observation window, myocardial tissue had not yet developed characteristic fibrotic pathological changes, precluding effective assessment of SQG’s preventive intervention effects on fibrosis.

MMP9 participates in ECM remodeling and regulates myocardial fibrosis. Our existing experimental data, derived from HE staining, TTC staining, and Western blot analyses, have sufficiently validated the central conclusion that SQG ameliorates acute-phase myocardial infarct size, relieves cardiomyocyte edema and structural disorganization, and suppresses inflammation-related signaling pathways through downregulation of MMP9/ADH1C. The primary constituents of SQG, flavonoids, saponins, and phytosterols, exert anti-inflammatory, antioxidant, and immunomodulatory effects that manifest over extended periods. We hypothesize that prolonged administration during the late IR injury phase (e.g., 1–4 weeks) may further improve myocardial remodeling and functional recovery through sustained downregulation of MMP9/ADH1C, modulation of the immune microenvironment, and inhibition of myocardial fibrosis. We fully acknowledge the critical role of myocardial fibrosis in the long-term prognosis of MIRI and have designated this as a priority for future research. We plan to conduct long-term intervention experiments (extending the administration period to 4 weeks) and systematically analyze the regulatory effects of SQG on post-MIRI myocardial fibrosis progression using Masson’s trichrome staining and collagen content assays, thereby completing the mechanistic chain linking acute-phase injury repair to long-term fibrosis modulation.

## 4. Materials and Methods

### 4.1. Reagents and Experimental Animals

Shenqi granules (SQGs) were sourced from Taiji Group Co., Ltd. in Chongqing, China. The HPLC chemical fingerprint pattern of SQG is provided in the Supplementary Materials. Isosorbide dinitrate tablets (ISDN) were obtained from Tianjin Pacific Pharmaceutical Co., Ltd. in Tianjin, China.

Male Sprague-Dawley rats (250–280 g) came from the Laboratory Animal Center of Shanxi Medical University. The experiments followed the “Laboratory Animal Care and Use Guidelines” and were approved by the Animal Ethics Committee of Shanxi University of Chinese Medicine (Approval No. AWE202503174) on 10 March 2025. Rats were housed in a controlled environment (25 ± 2 °C, 60 ± 5% humidity, 12 h light/dark cycle) with free access to food and water for a week.

### 4.2. Establishment of MIRI Animal Model and Administration Regimen

SQG is an over-the-counter (OTC) traditional Chinese medicine approved by the National Medical Products Administration. Its label specifies a clear daily human dose that has already been vetted for both safety and efficacy in widespread clinical use. The medium rat dose was calculated with the body-surface-area (BSA) method recommended in Pharmacological Experimental Methodology: rat dose = human dose × 6.25 × (rat BSA/human BSA). Using a 60 kg adult (BSA ≈ 1.62 m^2^) and a 200 g rat (BSA ≈ 0.04 m^2^), the labeled human daily dose of 10 g converts to ≈ 1.5 g/kg in the rat. This medium dose served as the pivotal intervention level. Low (0.75 g/kg) and high (3.0 g/kg) levels are simple ½× and 2× multiples of the medium dose, a conventional scheme for herbal preparations that readily reveals dose–response relationships.

The Sprague-Dawley rats were randomly divided into six groups (ten rats per group): a (1) sham-operated group; (2) an I/R group; (3) an I/R with positive drug treatment (ISDN, 5 mg/kg) group; (4) an I/R with low-dose SQG treatment (SQG-L, 0.75 g/kg) group; (5) an I/R with moderate-dose SQG treatment (SQG-M, 1.5 g/kg) group; and (6) an I/R with high-dose SQG treatment (SQG-H, 3.0 g/kg) group. The treatment groups were given a once-daily intragastric (i.g.) administration of SQG at different doses for 7 consecutive days starting from 7 days before ischemia modeling. At the same time, the sham and model groups were administered the same volume of normal saline aqueous solution.

Twelve hours before modeling, rats were food-deprived but allowed free water access. One hour after the last drug administration, anesthesia was induced via intraperitoneal sodium pentobarbital injection. Needle electrodes were subcutaneously inserted into the limbs, connected to an electrocardiograph (ECG) physiological recorder to continuously monitor lead II ECG. A midline anterior cervical incision was made, followed by tracheostomy and tube insertion linked to an animal ventilator. After thoracotomy, the fibrous pericardium was cut open, and a rib spreader was placed. The left anterior descending coronary artery (LAD) was precisely ligated between the left atrial appendage and pulmonary artery trunk using a suture to induce myocardial ischemia. Successful ligation was confirmed when the anterior wall distal to the ligation site turned purple, and the ECG showed significant ST-segment elevation. After 2 h, the ligature was released to restore LAD blood flow. After 24 h of reperfusion, the rats were euthanized.

### 4.3. Electrocardiograph (ECG)

An electrocardiogram (limb lead II) was recorded using a Bio Amp signal amplifier (ADInstruments, Dunedin, New Zealand) and a multi-channel physiological recorder (ADInstruments, Dunedin, New Zealand). The ECG parameters monitored included PR interval, QRS interval, QT interval, corrected QT interval (QTc), T wave, ST-segment, and heart rate.

### 4.4. 2,3,5-Triphenyltetrazolium Chloride (TTC) Staining

Remove the fresh heart and freeze it at −20 °C for 15 min. Cut the heart evenly into five slices from the apex to the coronary artery ligation site. Stain the slices with 2% TTC solution at 37 °C in the dark for 30 min, then rinse them with normal saline. Normal myocardial areas turn bright brick-red, while necrotic areas, lacking activity, stay uncolored. To preserve the slices’ shape and structure, fix them in 10% formalin for 24 h. Use Image J2x software for analysis. Assess the severity of MI by calculating the ratio of the necrotic area to the total area.

### 4.5. Hematoxylin and Eosin (HE) Staining

HE staining of tissue sections was performed as previously described to evaluate cellular morphology and tissue organization [50].

### 4.6. Network Pharmacology and Enrichment Analysis

The gene expression matrix GSE62646 was obtained from the GEO database, and samples were divided into control and patient groups. Data were filtered by removing rows (genes) and columns (samples) where the proportion of NA values exceeded 50%. Missing data were imputed using the impute.knn function from the R package impute with K-nearest neighbors. Differential gene analysis was done via the R package limma (version 3.40.6) using generalized linear models. The lmFit function was used for multiple linear regression modeling, followed by empirical Bayes moderation with eBayes to compute corrected *t*-statistics, F-statistics, and log-odds of differential expression. Genes with fold change ≥ 1.3 and adjusted *p*-value < 0.05 were considered differentially expressed.

The Traditional Chinese Medicine Systems Pharmacology platform was used to identify chemical components of *Astragalus membranaceus* and *Codonopsis pilosula* [51]. Active compounds were selected based on OB ≥ 30% and DL ≥ 0.18. Targets of these compounds were extracted, and their Gene Symbols were found using the STRING database. A Sankey diagram was created to visualize the “herb-active component-target” relationships.

Venny 2.0.1 was used to analyze the intersection genes among different analysis groups. GO and KEGG enrichment analyses were performed with the R package clusterProfiler (Version 4.14.3).

### 4.7. Assessment of the Immune Landscape

CIBERSORT and Xcell algorithms were used to analyze immune cell infiltration differences between ischemic heart patients and normal controls. Cell subgroups with significant differences were found using a rank-sum test.

### 4.8. Weighted Gene Co-Expression Network Analysis (WGCNA)

In constructing co-expression networks and partitioning modules, we first preprocessed gene expression data, calculating each gene’s median absolute deviation (MAD) and removing the bottom 50% with the lowest MAD to filter out low-variance genes. Then, the goodSamplesGenes function from the R package WGCNA was utilized to detect and remove outlier genes and samples, ensuring data quality. Based on the preprocessed data, a scale-free co-expression network was built with WGCNA. Initially, we calculated the Pearson correlation matrix for all gene pairs and performed preliminary clustering with average linkage. Subsequently, a weighted adjacency matrix was constructed using a power function, with β set to 18 to meet the scale-free network criteria through multiple tests.

The adjacency matrix was converted into a Topological Overlap Matrix (TOM) to measure gene network connectivity, with a gene’s connectivity defined as the sum of its adjacencies to all other genes. A dissimilarity matrix (1-TOM) was computed, and average linkage hierarchical clustering based on TOM dissimilarity was applied to group genes, with a minimum module size of 30 genes. During module partitioning, the sensitivity parameter was set to 3 to enhance clustering. Module eigengene dissimilarity was computed, modules with a dendrogram cut height of 0.25 were merged, and co-expression modules were obtained. Module membership (MM), the correlation between module eigengenes and gene expression, was calculated to identify core genes in clinically significant modules. Genes with |MM| > 0.8 were identified as high-connectivity hub genes from modules highly correlated with grouping, serving as key targets for subsequent analysis.

### 4.9. Receiver Operating Characteristic (ROC) Analysis

ROC analysis was performed using the R package pROC (version 1.17.0.1). The roc function from this package was applied to analyze the GSE62646 dataset, and the ci function was used to evaluate the AUC and its confidence intervals, thereby generating the AUC results.

### 4.10. Molecular Docking

Molecular docking analysis was performed based on a previous research method [52]. Subject the two proteins (ADH1C and MMP9) and six compounds (hederagenin, 7-O-methylisomucronulatol, luteolin, quercetin, stigmasterol, and (6aR,11aR)-9,10-dimethoxy-6a,11a-dihydro-6H-benzofurano[3,2-c]chromen-3-ol) to blind docking using cb-dock2 (https://cadd.labshare.cn/cb-dock2, accessed on 11 March 2026).

### 4.11. Molecular Dynamics Simulation

Molecular dynamics simulations were conducted as per the method outlined by Liu et al. [52]. The simulation setup involved selecting molecules, placing them in a simulation box, and adding water and ions. Force field parameters were assigned, followed by energy minimization and equilibration steps (NVT and NPT). For the production run, parameters including a time step of 2 fs, a total simulation time of 100 ns, and data output frequency were specified. The results were analyzed using metrics such as Root Mean Square Deviation (RMSD), Root Mean Square Fluctuation (RMSF), Radius of Gyration (Rg), and Solvent Accessible Surface Area (SASA), and visualizations were created through images and animations.

### 4.12. Western Blot

Western blot analysis was carried out as described by Wen et al. [53]. Primary antibodies used were against MMP9 (1:1000, Proteintech, Wuhan, China), ADH1C (1:1000, Proteintech, China), and GAPDH (1:2000, Proteintech, China). For detection, a horseradish peroxidase (HRP)-linked goat anti-rabbit/mouse secondary antibody (1:5000, Proteintech, China) was utilized.

### 4.13. Statistical Analyses

All results are expressed as the mean ± standard error of the mean (SEM) to reflect data variability. The study’s primary objective was to compare population means among multiple groups. Normality was confirmed for every continuous variable by the Shapiro–Wilk test (*p* > 0.05). Homogeneity of variances was verified with Levene’s test (*p* > 0.05). When both assumptions were satisfied, one-way ANOVA followed by Tukey’s post hoc test was used for multiple comparisons; otherwise, the Kruskal–Wallis H test with Dunn’s correction was applied. # indicates comparison with the Sham group (# *p* < 0.05, ## *p* < 0.01, ### *p* < 0.001), while * indicates comparison with the I/R model group (* *p* < 0.05, ** *p* < 0.01, *** *p* < 0.001). All analyses were performed with GraphPad Prism 9.0.

## 5. Conclusions

In conclusion, this study elucidates the protective effects of SQG against MIRI through an integrated network pharmacology and experimental validation approach. Our findings demonstrate that SQG significantly reduces myocardial infarct volume, preserves cardiac function, and ameliorates histopathological damage in MIRI rats. Mechanistically, these beneficial effects are attributed to SQG’s capacity to downregulate the expression of MMP9 and ADH1C, thereby attenuating inflammatory responses, suppressing extracellular matrix degradation, and modulating immune–metabolic crosstalk within the cardiac microenvironment. The identification of MMP9 and ADH1C as core therapeutic targets, combined with the favorable binding profiles of SQG’s key bioactive constituents (luteolin and stigmasterol), provides a molecular basis for understanding the multi-component, multi-target characteristics of this traditional Chinese medicine formula. These results position SQG as a promising therapeutic candidate for myocardial infarction, offering potential advantages in acute cardioprotection and long-term cardiac remodeling prevention. Future investigations should extend to chronic administration paradigms to evaluate SQG’s efficacy in modulating post-infarction fibrosis and complete the mechanistic trajectory from acute injury repair to long-term cardiac recovery.

## Data Availability

The original contributions presented in this study are included in the article/Supplementary Material. Further inquiries can be directed to the corresponding authors.

## Supplementary Materials

The following supporting information can be downloaded at: https://www.mdpi.com/article/10.3390/ph19030475/s1, Table S1: 43 active compounds from Astragali Radix and Codonopsis Radix.

## Abbreviations

ADH1C, alcohol dehydrogenase type 1C; AUC, Area Under the Curve; BP, biological process; CC, cellular component; DEGs, differentially expressed genes; ECG, electrocardiograph; GO, gene ontology; GS, gene significance; HE, hematoxylin and eosin; HRP, horseradish peroxidase; ISDN, isosorbide dinitrate tablets; I/R, ischemia–reperfusion; KEGG, Kyoto Encyclopedia of Genes and Genomes; LAD, left anterior descending coronary artery; MAD, median absolute deviation; MF, molecular function; MI, myocardial infarction; MIRI, myocardial ischemia–reperfusion injury; MM, module membership; MMP-9, Matrix metalloproteinase-9; PCI, percutaneous coronary intervention; Rg, Radius of Gyration; RMSD, Root Mean Square Deviation; RMSF, Root Mean Square Fluctuation; ROC, receiver operating characteristic; SASA, Solvent Accessible Surface Area; SEM, standard error of the mean; SQG, Shenqi granules; TCM, traditional Chinese medicine; TCMSP, Traditional Chinese Medicine Systems Pharmacology; TOM, Topological Overlap Matrix; TTC, 2,3,5-Triphenyltetrazolium chloride; WGCNA, weighted gene co-expression network analysis
