# Supplementary material for: Shenqi Granules Enhance Recovery from Myocardial Ischemia–Reperfusion Injury by Downregulating MMP9 and ADH1C
Source: Pharmaceuticals (Basel). 2026 Mar 13;19(3):475. doi: 10.3390/ph19030475 (PMC13029134; doi:10.3390/ph19030475)
Supplement: Supplementary file 1 [file pharmaceuticals-19-00475-s001.zip › pharmaceuticals-4180230-supplementary.pdf]

Table S1. 43 active compounds from Astragali Radix and Codonopsis Radix

Mol ID	Molecule Name	MW	AlogP	Hdon	Hacc	OB (%)	Caco-2	BBB	DL	FASA-	HL
MOL000033	(3S,8S,9S,10R,13R,14S,17R)-10,13-dimethyl-17-[(2R,5S)-5-propan-2-yloctan-2-yl]-2,3,4,7,8,9,11,12,14,15,16,17-dodecahydro-1H-cyclopenta[a]phenanthren-3-ol	428.82	8.54	1	1	36.23	1.45	1.09	0.78	0	5.22
MOL000296	hederagenin	414.79	8.08	1	1	36.91	1.32	0.96	0.75	0	5.35
MOL000378	7-O-methylisomucronulatol	316.38	3.38	1	5	74.69	1.08	0.84	0.3	0	2.98
MOL000371	3,9-di-O-methylnissolin	314.36	2.89	0	5	53.74	1.18	0.63	0.48	0	9
MOL000380	(6aR,11aR)-9,10-dimethoxy-6a,11a-dihydro-6H-benzofurano[3,2-c]chromen-3-ol	300.33	2.64	1	5	64.26	0.93	0.55	0.42	0	8.49
MOL000211	Mairin	456.78	6.52	2	3	55.38	0.73	0.22	0.78	0.26	8.87
MOL000392	formononetin	268.28	2.58	1	4	69.67	0.78	0.02	0.21	0	17.04
MOL000442	1,7-Dihydroxy-3,9-	314.31	3.11	2	6	39.05	0.89	-0.04	0.48	0	7.95

Mol ID	Molecule Name	MW	AlogP	Hdon	Hacc	OB (%)	Caco-2	BBB	DL	FASA-	HL
	dimethoxy pterocarpene										
MOL000387	Bifendate	418.38	2.56	0	10	31.1	0.15	-0.06	0.67	0	17.96
MOL000239	Jaranol	314.31	2.09	2	6	50.83	0.61	-0.22	0.29	0.29	15.5
MOL000417	Calycosin	284.28	2.32	2	5	47.75	0.52	-0.43	0.24	0	17.1
MOL000354	isorhamnetin	316.28	1.76	4	7	49.6	0.31	-0.54	0.31	0.32	14.34
MOL000422	kaempferol	286.25	1.77	4	6	41.88	0.26	-0.55	0.24	0	14.74
MOL000098	quercetin	302.25	1.5	5	7	46.43	0.05	-0.77	0.28	0.38	14.4
MOL000379	9,10-dimethoxypterocarpan-3-O- β -D-glucoside	462.49	0.74	4	10	36.74	-0.63	-1.5	0.92	0	13.06
MOL000433	FA	441.45	0.01	7	13	68.96	-1.5	-2.59	0.71	0	24.81
MOL000439	isomucronulatol-7,2'-di-O-glucosiole	626.67	-0.68	8	15	49.28	-2.22	-3.36	0.62	0	0.93
MOL008407	(8S,9S,10R,13R,14S,17R)-17-[(E,2R,5S)-5-ethyl-6-methylhept-3-en-2-yl]-10,13-dimethyl-1,2,4,7,8,9,11,12,14,15,16,17-dodecahydrocyclopenta[a]phenanthren-3-one	410.75	7.31	0	1	45.4	1.49	1.26	0.76	0.24	5.65

Mol ID	Molecule Name	MW	AlogP	Hdon	Hacc	OB (%)	Caco-2	BBB	DL	FASA-	HL
MOL001006	poriferasta-7,22E-dien-3beta-ol	412.77	7.64	1	1	42.98	1.45	1.11	0.76	0.21	5.48
MOL007514	methyl icosa-11,14-dienoate	322.59	7.55	0	2	39.67	1.47	1.1	0.23	0.18	5.24
MOL004355	Spinasterol	412.77	7.64	1	1	42.98	1.44	1.04	0.76	0.21	5.32
MOL006774	stigmast-7-enol	414.79	8.08	1	1	37.42	1.39	1.04	0.75	0.22	6.28
MOL000449	Stigmasterol	412.77	7.64	1	1	43.83	1.44	1	0.76	0.22	5.57
MOL003036	ZINC03978781	412.77	7.64	1	1	43.83	1.32	0.96	0.76	0	5.79
MOL003896	7-Methoxy-2-methyl isoflavone	266.31	3.36	0	3	42.56	1.16	0.56	0.2	0.33	16.89
MOL005321	Frutinone A	264.24	2.7	0	4	65.9	0.89	0.46	0.34	0.47	19.1
MOL002879	Diop	390.62	7.44	0	4	43.59	0.79	0.26	0.39	0.28	3.6
MOL002140	Perlolyrine	264.3	3.2	2	3	65.95	0.88	0.15	0.27	0.21	12.62
MOL008397	Daturilin	436.64	4.34	0	4	50.37	0.61	0.06	0.77	0.32	5.73
MOL008411	11-Hydroxyrankinidine	356.46	1.04	2	6	40	0.29	-0.19	0.66	0.28	10.8
MOL008400	glycitein	284.28	2.32	2	5	50.48	0.56	-0.29	0.24	0.3	16.32
MOL007059	3-beta-Hydroxymethyllenetanshiquinone	294.32	3.16	1	4	32.16	0.38	-0.48	0.41	0.36	22.51
MOL000006	luteolin	286.25	2.07	4	6	36.16	0.19	-0.84	0.25	0.39	15.94
MOL008393	7-(beta-Xylosyl)cephalomannine_qt	830.02	3.21	4	14	38.33	-0.87	-1.59	0.29	0	5.97
